# Glycated hemoglobin independently or in combination with fasting plasma glucose versus oral glucose tolerance test to detect abnormal glycometabolism in acute ischemic stroke: a Chinese cross-sectional study

**DOI:** 10.1186/s12883-014-0177-0

**Published:** 2014-09-12

**Authors:** Shuolin Wu, Yuzhi Shi, Yuesong Pan, Jingjing Li, Qian Jia, Ning Zhang, Xingquan Zhao, Gaifen Liu, Yilong Wang, Yongjun Wang, Chunxue Wang

**Affiliations:** Department of Geriatrics, Beijing Tiantan Hospital, Capital Medical University, Beijing, China; Department of Neurology, Beijing Tiantan Hospital, Capital Medical University, Beijing, China

**Keywords:** Diabetes mellitus, HbA1c, Ischemic stroke, OGTT, Prediabetes

## Abstract

**Background:**

The investigation of glycated hemoglobin (HbA1c) as a diagnostic tool for abnormal glycometabolism is lack in acute ischemic stroke patients in China and worldwide. This paper was aimed to determine whether HbA1c, fasting plasma glucose (FPG), or HbA1c combined with FPG, could be used to screen for diabetes mellitus (DM) or prediabetes in acute ischemic stroke patients without previous DM.

**Methods:**

Acute ischemic stroke patients without previous DM (n = 1,316) were selected from the Abnormal gluCose Regulation in Patients with Acute StrOke acrosS China Study (ACROSS-China). Oral glucose tolerance test (OGTT), HbA1c, FPG, and HbA1c combined with FPG were used as the screening methods to categorize the glycometabolic status. OGTT was taken as the golden method. Venn diagrams and the overlap index were used to determine the associations among the three methods of identifying abnormal glycometabolism. The area under the receiver operating characteristic curve (AUROC) and Youden index were used to assess and compare the accuracy in detecting abnormal glycometabolism. Youden analyses were performed to determine the ideal cutoff values of HbA1c in diagnosing abnormal glycometabolism.

**Results:**

In acute ischemic stroke patients without previous DM, the overlaps of HbA1c versus OGTT, HbA1c versus FPG, and all the three methods independently, were low for detecting abnormal glycometabolism (all <50%). HbA1c can significantly detect more cases of prediabetes than OGTT (P < 0.001). The combination of HbA1c and FPG significantly raised the sensitivity to over 60.0%, specificity to over 80.0%, and the diagnostic accuracy (Youden index from under 40.0% to 42.4%)for DM. HbA1c of 5.7%-6.4% had a low to moderate concordance with OGTT for identifying prediabetes (AUROC = 0.557, P = 0.001). HbA1c values of 6.3% and 5.9% were found to be the ideal cutoff values for detecting DM and abnormal glycometabolism in our data, respectively.

**Conclusions:**

The combination of HbA1c and FPG increased the diagnostic rate of DM when compared with OGTT, and increased the diagnostic accuracy for DM compared with HbA1c or FPG alone. Our results advocate the use of HbA1c as screening tool for the diagnosis of pre-diabetes.

**Electronic supplementary material:**

The online version of this article (doi:10.1186/s12883-014-0177-0) contains supplementary material, which is available to authorized users.

## Background

Glycated hemoglobin (HbA1c) can be indicative of the average glucose level of the preceding 2–3 months. HbA1c is an easy method to screen for diabetes mellitus (DM) but compared to oral glucose tolerance test (OGTT), its sensitivity for detecting DM is low. However, the American Diabetes Association (ADA) endorses the use of HbA1C based on its familiarity to clinicians, simple manipulation, and no need to fast. Meanwhile, an HbA1c level of 5.7-6.4% was recommended as the diagnostic criterion for prediabetes due to a high risk for developing DM from the patients with this HbA1c level [1,2].

The test of fasting plasma glucose (FPG) requires no caloric intake for at least eight hours. FPG has been typically used to monitor the glucose status of patients in clinics or hospitals. The benefits of using FPG include its simplicity of use, low cost, and ease of interpretation. However, its results are easily influenced by fluctuations in glucose level [3].

According to the World Health Organization (WHO) criteria in 1997, OGTT is one method to diagnose abnormal glucose levels. OGTT following an overnight fast for at least eight hours was performed via an oral intake of a standard dose of 75 g anhydrous glucose dissolved in water. Fasting plasma glucose levels were measured prior to administering the anhydrous glucose and postprandial glucose was evaluated two hours later. Patients were not allowed or recommended to have a special diet during the two hours. Fasting plasma glucose and 2-h plasma glucose (PG2h) were used in combination to diagnose DM or prediabetes [4,5].

Since a high prevalence of abnormal glycometabolism diagnosed using OGTT among Chinese patients with acute stroke has been estimated [6] and both DM and a high ‘normal’ glycamia status are high risks for stroke [7], there is a great need to find an efficient screening test to identify abnormal glycometabolism in patients with acute ischemic stroke. However, OGTT is limited in the clinical practice because it is inconvenient, time-consuming, relatively expensive, and requires the patient to fast [3]. A method aside from OGTT is needed to diagnose abnormal glycometabolism quickly and easily.

Up to date, although several recent studies worldwide have compared the ability of diagnosing abnormal glycometabolism between OGTT and HbA1c with or without FPG based on disease spectrums and patient populations [8–12], the results were inconsistent and a similar study focus on acute ischemic stroke patients was still lacking [13].

In the present study, we aimed to compare the diagnostic accuracy among HbA1c, FPG, and OGTT for newly-diagnosed DM and pre-diabetes among patients with acute ischemic stroke. An ideal cut-off value of HbA1c was also pursued to better diagnose abnormal glycometabolism. HbA1c or combining HbA1c and FPG was hypothesized to be a better method than OGTT for screening abnormal glycometabolism.

## Methods

### Subjects

**Figure 1:**
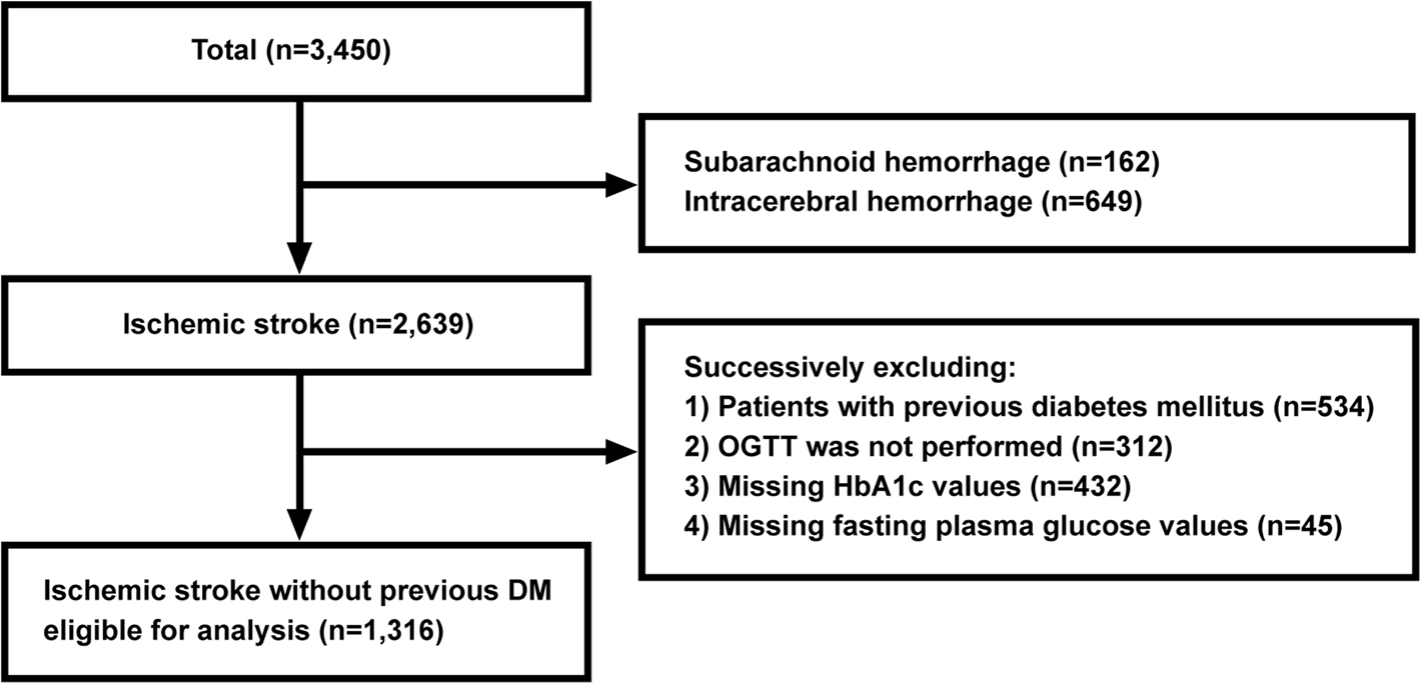
**Flow chart of patient selection.**

Patients (n = 1,316) were selected from the ACROSS-China study (n = 3,450) [6]. Patients were excluded successively as below: subarachnoid hemorrhage (n = 162), intracerebral hemorrhage (n = 649), previous diagnosis of DM (n = 534), patients who declined OGTT (n = 312), missing HbA1c value (n = 432), and missing FPG value (n = 45). OGTT, HbA1c, FPG, and HbA1c combined with FPG were used to categorize the glycometabolism status in patients with acute ischemic stroke (Figure 1).

### Diagnostic criteria and methods of abnormal glycometabolism status

Blood samples were collected for the evaluation of FPG and HbA1c within 24 hours after admission. OGTT was done on the 14th day after stroke onset.

The WHO 1999 criteria for OGTT to identify DM, prediabetes, an normoglycemia were as follows: 1) DM defined as FPG ≥7.0 mmol/L and/or PG2h ≥11.1 mmol/L; 2) intermediate hyperglycaemia, which consists of impaired glucose tolerance (IGT) (FPG <7.0 mmol/L and PG2h between 7.8 mmol/L and 11.1 mmol/L), and impaired fasting glucose (IFG) (FPG between 6.1 mmol/L and 7.0 mmol/L, and PG2h <7.8 mmol/L); and 3) normoglycemia defined as FPG <6.1 mmol/L and a PG2h <7.8 mmol/L. All the patients were categorized into these three groups according to OGTT results.

The FPG criteria for diagnosing glycometabolism status were as follows: DM defined as ≥7.0 mmol/L, prediabetes was defined as from 5.6 to 6.9 mmol/l [4,5]. Patients who had FPG of ≥7.0 mmol/L were required to repeat FPG test on the next day. A diagnosis of DM was made for a patient only when both FPG values were ≥7.0 mmol/L. When the FPG value was ≥7.0 mmol/L at the first time but 6.1 - 7.0 mmol/L at the second time, a diagnosis of prediabetes was made. When the FPG value was ≥7.0 mmol/L at the first time but <5.6 mmol/L at the second time, normoglycaemia was diagnosed for the patient.

The criteria of HbA1c were used to predict DM, prediabetes, and normoglycemia were: HbA1c ≥6.5%, HbA1c5.7-6.4%, and HbA1c <5.7% were used as the criteria for diabetes, prediabetes, and normoglycemia [1,2].

The measurement methods of OGTT and HbA1c were the same as described in previously published studies [6,7]. The blood samples for HbA1c evaluation were separated and the plasmas were properly processed, refrigerated at −20°C, and transported to Beijing Tiantan Hospital in Beijing, China. The laboratory in Beijng Tiantan Hospital was certified by the National Glycohemoglobin Standardization Program (NGSP) for HbA1c measurement. HbA1c was measured using ‘high performance liquid chromatographic analysis’ (HPLC) by a Bio-Rad VariantIIanalyzer (Bio-Rad Laboratories, Hercules, CA) with a reference value of 4.1-6.5% in accordance with the standard in the Diabetes Control and Complications Trial (DCCT) and NGSP. The intra-assay coefficient of variation (CV) was 2.5% and the interassay CV was <4.0%, both of them were within the limits of the NGSP.

### Other variables

Clinical data from patients were obtained within 24 hours after admission. Information included age, gender, smoking status (current smoking was defined as an individual who smoked at the time of stroke), alcohol intake (moderate or severe drinking was defined as the consumption of at least two standard alcoholic beverages per day), a previous medical history, body mass index (BMI), blood routine and biochemical indexes including blood cell counts, hemoglobin, platelet counts, creatinine, triglyceride, lipoprotein, and cholesterol levels.

### Ethics statement

Procedures at all participating centers were approved by the ethics committee at Beijing Tiantan Hospital. Written informed consent was obtained from all patients, or from their designated family members.

### Statistical analysis

Clinical data of patients with ischemic stroke without previous DM were compared. Study patients were separated based on the new glycometabolic status according to OGTT results. Continuous data and categorical data were presented as mean ± standard deviation and frequency/ratio, respectively. The variables were compared through one-way analysis of variance, test χ2 or Fisher’s exact test. Detection rate of abnormal glycometabolism diagnosis were calculated using various diagnostic methods. Venn diagrams were used to illustrate the concordances and variances among the three methods of detecting the abnormal glycometabolism. The overlap index was calculated as the event number of concordant diagnoses divided by the event number diagnosed by either HbA1c or FPG, either HbA1c or OGTT, and either HbA1c or FPG or OGTT, which reflected the agreements among the three methods. An overlap index value of lower than 50% was considered poor agreement. The receiver operating characteristic curve was used to calculate the area under the receiver operating characteristic curve (AUROC), sensitivity, and specificity. OGTT was taken as the golden method in AUROC analysis. Youden indexes (maximum values of sensitivity plus specificity minus one) of the three tests were compared for diagnostic accuracy of identifying abnormal glycometabolism. Youden analysis was done to discover an ideal cut-off value of HbA1c for diagnosing abnormal glycometabolism status in our data. Because both HbA1c and FPG tests were taken in the same set of subjects, the AUROCs of the two tests we got were not independent. The comparisons between the AUROCs were performed by using the nonparametric Z test with the correlation between AUROCs taken into account [14]. The calculating equation was $$ `\mathrm{Z}=\frac{\mathrm{A}1-\mathrm{A}2}{\sqrt{\mathrm{SE}1*\mathrm{SE}1+\mathrm{SE}2*\mathrm{SE}2\hbox{-} 2*\mathrm{r}*\mathrm{SE}1*\mathrm{SE}2}}' $$, ‘r’ was calculated manually according to the method described in the published [14]. The equation ‘r = (r_n_ + r_a_)/2’ was used, r_n_ indicates the Kendall tau correlation coefficient between different diagnostic tools in the ‘no disease’ group, r_a_ indicates the Kendall tau correlation coefficient between different diagnostic tools in the ‘disease’ group. SPSS 19.0 software (SPSS Inc., Chicago, IL) was used to perform all the other analyses. A two-tailed P <0.05 was considered statistically significant.

## Results

### Clinical characteristics according to glycometabolism status by OGTT

There were 1,316 patients included in the present analysis and their average age was 62.4 years old and 63.3% of them were male. Current smokers occupied 33% and moderate to severe drinkers occupied 15.5%. The mean systolic blood pressure was over 140 mmHg. More than 50% of the patients had a history of hypertension. The mean levels of FPG and HbA1c were 5.7 mmol/L and 6.0%, respectively. The mean level of low-density lipoprotein was over 3.0 mmol/L.

The mean age, FPG, HbA1c, white cell counts, insulin resistance index, and low-density lipoprotein were all higher in the DM group than those in the prediabetes and normoglycemia groups (all P < 0.05). The differences of other variables among the groups were not statistically significant (see Additional file 1).

### Detection of newly-diagnosed abnormal glycometabolic status by HbA1c, FPG, and OGTT

The three tests identified different patients with abnormal glycometabolic status among ischemic stroke patients without previous DM (Table 1). There were 356 DM and 425 prediabetes cases categorized by OGTT. HbA1c identified 327 DM and 450 prediabetes cases, and FPG identified 188 DM and 333 prediabetes cases.

**Table 1:** **Glycometabolic status categorized by HbA1c, FPG and OGTT**

				**OGTT**	
		**Total (n = 1,316)**	**DM (n = 356)**	**Prediabetes (n = 425)**	**Normoglycemia (n = 535)**
HbA1c^a^	DM	327	188	84	55
	Prediabetes	450	105	178	167
	Normoglycemia	539	63	163	313
FPG^b,c^	DM	188	130	38	20
	Prediabetes	333	123	116	94
	Normoglycemia	795	103	271	421

^a^and ^b^indicate P < 0.05 when the glycometabolic status was compared with the diagnosis by OGTT.

^c^indicates P < 0.05 when the glycometabolic status was compared with the diagnosis by HbA1c. HbA1c indicates glycated hemoglobin; FPG indicates fasting plasma glucose.

OGTT detected more patients with DM than HbA1c (27.1% vs. 24.8%, P < 0.001), while HbA1c detected more prediabetes than OGTT (34.2% vs. 32.3%, P < 0.001). Among the three tests, FPG had the lowest detection rate for DM (14.3%) and for prediabetes (25.3%), respectively.

**Figure 2:**
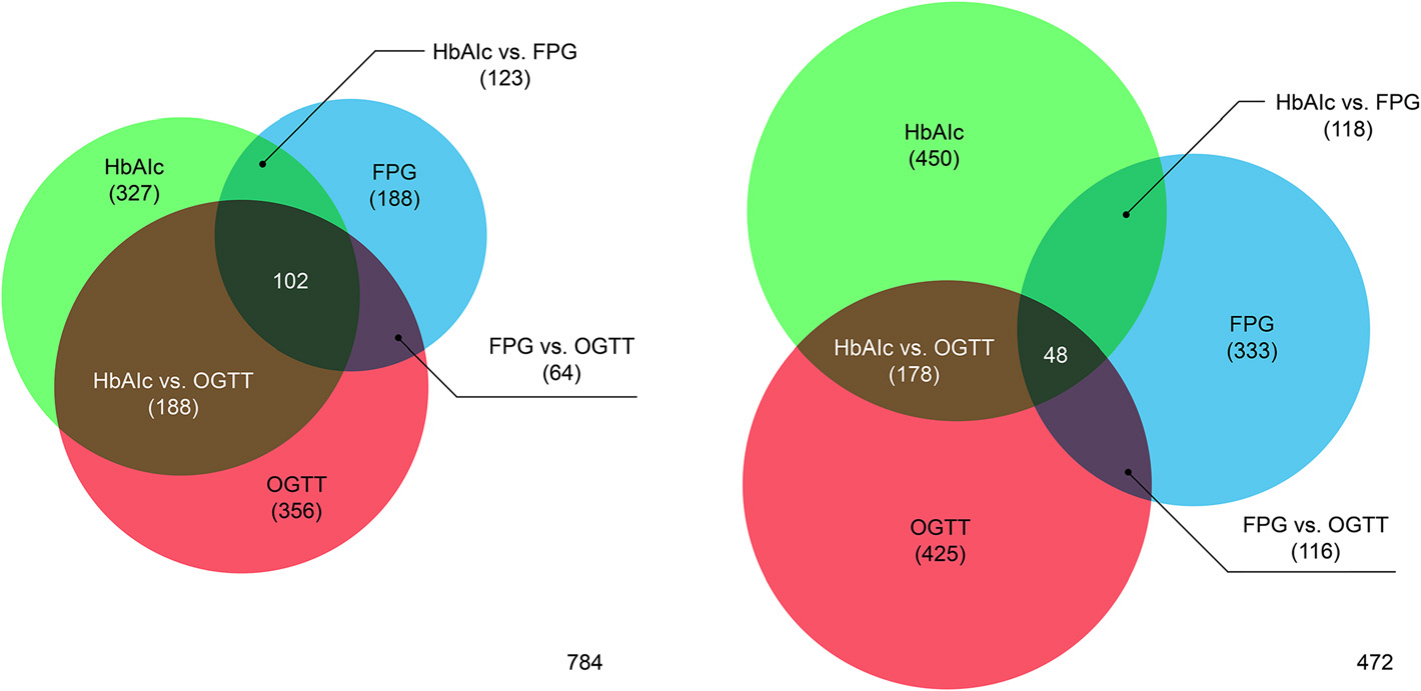
**Venn Diagrams among HbA1c, FPG and OGTT for detecting newly-diagnosed abnormal glycometabolism.** The figure was used to illustrate the concordances and variances between the three methods of detecting the abnormal glycometabolism in acute ischemic stroke patients without previous diabetes mellitus (IS without previous DM). DM: the newly-diagnosed diabetes mellitus patients. preDM: the newly-diagnosed prediabetes patients. Number in bracket: the number of the patients. Box in grey: the total patients (n = 1316). Circle in green: the patient number of DM or prediabetes detected by HbA1c. Circle in blue: the patient number of DM or prediabetes detected by FPG. Circle in red: the patient number of DM or prediabetes detected by OGTT. HbA1c vs. FPG: the concordance diagnosed patients of DM or prediabetes detected by HbA1c and FPG. HbA1c vs. OGTT: the concordance diagnosed patients of DM or prediabetes detected by HbA1c and OGTT. FPG vs. OGTT: the concordance diagnosed patients of DM or prediabetes detected by FPG and OGTT.

The Venn diagrams illustrated the association among the three tests (Figure 2). 102 DM (19.2%) and 48 prediabetes (5.7%) cases were concordantly detected by all the three tests. HbA1c and OGTT concordantly detected 188 DM (38.0%) and 178 prediabetes (25.5%) patients. HbA1c and FPG concordantly detected 123 DM (31.4%) and 118 prediabetes (17.7%) patients.

### Comparison of the accuracy for diagnosing newly-diagnosed abnormal glycometabolism between HbA1c/FPG and the combined use (OGTT as the golden method)

Compared with HbA1c or FPG alone, the combination of them increased the AUROC of diagnosing DM from 0.692 (HbA1c) and 0.652 (FPG) to 0.712 (the combination) (AUC comparison between HbA1c and the combination, Z = 1.78 < 1.96, P > 0.05; AUC comparison between FPG and the combination, Z = 1.72 < 1.96, P > 0.05). Youden index of the combination was higher than either of them (Table 2).

**Table 2:** **HbA1c and FPG versus OGTT in diagnosing abnormal glycometabolic status**

		**AUROC**	**Standard error**	**95% CI**	**P**	**Sensiti-vity**	**Specif-icity**	**Youden index**
HbA1c	DM	0.692	0.018	0.657-0.726	<0.001	0.528	0.855	0.383
	Prediabetes	0.557	0.017	0.523-0.590	0.001	0.419	0.695	0.114
	AGM	0.648	0.016	0.617-0.678	<0.001	0.711	0.585	0.296
FPG	DM	0.652	0.019	0.616-0.689	<0.001	0.365	0.940	0.305
	Prediabetes	0.515	0.017	0.481-0.548	0.388	0.273	0.756	0.029
	AGM	0.654	0.015	0.624-0.684	<0.001	0.521	0.787	0.308
HbA1c	DM	0.712	0.017	0.678-0.745	<0.001	0.607	0.817	0.424
With FPG	Prediabetes	0.554	0.017	0.521-0.587	0.001	0.579	0.530	0.109
	AGM	0.650	0.015	0.623-0.681	<0.001	0.812	0.488	0.300

HbA1c indicates glycated hemoglobin; FPG indicates fasting plasma glucose; OGTT indicates oral glucose tolerance test; AUROC indicates the area under the receiver operating characteristic curve; CI indicates confidence interval; AGM indicates abnormal glucose metabolism.

In diagnosing prediabetes, HbA1c had a low to moderate concordance with OGTT (AUROC:0.557, P = 0.001). FPG had a poor concordance with OGTT for detecting prediabetes (P = 0.388). The combination of HbA1c and FPG had a slightly weak concordance with OGTT when compared with HbA1c alone (AUROC: 0.557 vs. 0.554) (Table 2). However, such a slight difference could not be identified as statistically significant since the nonparametric Z test was not suitable (average Kendall tau correlation coefficient = 0.52, average AUC was 0.556 (<0.700), the correlation coefficient ‘r’ could not be looked up in Table I of the Hanley et al. study [14], thus, Z value could not be calculated).

The combination of HbA1c and FPG increased the sensitivity of detecting DM to 60.7%, and also increased the sensitivity of detecting prediabetes to 57.9% when comparing HbA1c or FPG alone (Table 2).

In comparing the specificity of HbA1c or FPG alone to the specificity of HbA1c and FPG combined for detecting DM, the specificity remained high. However, the specificity of detecting prediabetes when HbA1c and FPG were combined use was decreased to 53%.

The combination of HbA1c and FPG increased sensitivity to 81.2% for detecting overall abnormal glycometabolism when compared with either test, but the tradeoff was to have a low specificity (48.8%). Therefore, combining the two methods resulted in better diagnostic accuracy than HbA1c alone, but worse than FPG alone (Table 2).

### Youden analysis for determining the ideal cutoff of HbA1c for detecting abnormal glycometabolism

HbA1c was as a continuous variable entered into the ROC analysis and OGTT remained as the reference index (Table 3).

**Table 3:** **The Youden analysis for the HbA1c cut-off value of abnormal glycometabolism**

	**Sensitivity**	**Specificity**	**Youden index**	**HbA1c cut-off value** ^**a**^
Diabetes mellitus	0.579	0.824	0.403	6.3%
Diabetes plus prediabetes	0.590	0.742	0.332	5.9%

^a^indicates the criteria lower limit of HbA1c for detecting prediabetes.

A value of HbA1c equal to 6.3% was found to be the best cutoff value for detecting DM in our data based on the Youden index (Youden index = 0.403 vs. 0.383 when HbA1c = 6.3% vs. 6.5%).

A value of HbA1c equal to 5.9% was found to be the ideal cutoff value for detecting prediabetes (Youden index =0.332 vs. 0.296 as HbA1c ≥ 5.9% vs. ≥ 5.7%)

## Discussion

As far as we know, only Huisa et al. has reported HbA1c as a screening tool to detect a considerable diagnostic percentage of abnormal glycometabolism among patients with ischemic stroke, but it did not compare HbA1c with other diagnostic tools and did not report ideal cutoff values of HbA1c to identify DM and prediabetes [13]. The present study found that the newly-diagnosed DM or prediabetes by the three tests, HbA1c, FPG, and OGTT among patients with acute ischemic stroke were not fully concordant. The overlap between HbA1c and FPG, HbA1c and OGTT, or the three was low respectively, as all values were under 50%.

HbA1c, FPG, and the combination of them had different diagnostic results in diagnosing DM or prediabetes, respectively. HbA1c identified a higher diagnostic rate of prediabetes than OGTT. HbA1c alone and FPG alone had a low to moderate concordance with OGTT when diagnosing DM, prediabetes, or overall abnormal glycometabolism. One exception was that FPG alone was significantly discordant with OGTT in the diagnosis of prediabetes. When compared with HbA1c or FPG alone, the combination of HbA1c and FPG had the following results: 1) improved diagnostic accuracy and sensitivity, with high specificity persistence for diagnosing DM; 2) increased sensitivity but decreased specificity for detecting the overall abnormal glycometabolism; 3) did not enhance the diagnostic accuracy for prediabetes compared with HbA1c alone.

### Diagnosis of DM

The detection rate of DM by HbA1c was higher than that by FPG. This finding was inconsistent with other studies [2]. This may be because FPG is more affected by fluctuating glucose levels than HbA1c [3].

Although the diagnostic rate of DM identified by HbA1c was lower than that by OGTT, which was in line with other studies [9,15], HbA1c alone for diagnosing DM was confirmed to be feasible in our study, which was also confirmed by other studies although their study populations were diverse with ours [16–18]. The combination of HbA1c and FPG significantly raised the diagnostic accuracy for DM compared with HbA1c or FPG alone, which was also confirmed by Hjellestad et al. [8]. Although the difference between AUROC of HbA1c and that of the combination of HbA1c and FPG was slight and not statistically significant (Z = 1.78, P > 0.05), the AUROC was indeed changed (AUROC changed from 0.692 to 0.712) and a much bigger sample size might settle the puzzle.

### Diagnosis of prediabetes

The detection rate of prediabetes by HbA1c was higher than that by OGTT, which was in line with findings by Hjellestad, et al. [8]. However, this was inconsistent with Lorenzo et al. [19] and that from the National Health and Nutrition Examination Survey [20]. Lorenzo, et al. found that the HbA1c level of 5.7-6.4% had a low sensitivity in detecting prediabetes because a large number of prediabetes cases existed in clinical and epidemiological settings, where a significant proportion of individuals with HbA1c lower than 5.5% had either IFG or IGT. Thus, they did not recommend HbA1c as a sole screening tool for prediabetes. Obesity, age, and race were also elucidated in their studies, which influenced the results of HbA1c as a diagnostic tool for prediabetes. These differences could explain the different results between these studies and our current research. Moreover, our study patients as well as those in the study by Hjellestad, et al. were both highly selective of chronic glycemic-overload status, rather than the general population from clinical or epidemiological settings. HbA1c can be indicative of chronic hyperglycemia overload [21]. Lorenzo et al. also noted that HbA1c has less precise correlates of insulin resistance and secretion (the core of DM) than FPG and PG2h (OGTT results) [19], which suggested that insulin-resistance status was closely related to OGTT, and compared with OGTT. HbA1c could provide more other information, mainly chronic glycemia status (overload or not), in vivo rather than insulin-resistance status. Thus, a lower detection rate of prediabetes and a higher one of DM by OGTT, compared with those by HbA1c, were presented in our study. The higher detection rate of prediabetes by HbA1c might reflect a high chronic glycemic overload in patients with ischemic stroke.

The FPG of 5.6-6.9 mmol/L had a statistically significant discordance with OGTT, which may be because the mean level of FPG in the OGTT-diagnosed prediabetes arm was 5.4 mmol/l. This did not reach the lower limit of the IFG range for detecting prediabetes. This may explain the slight decrease in diagnostic accuracy for prediabetes when combining HbA1c and FPG.

Our results advocate the use of HbA1c as a screening tool for the diagnosis of prediabetes. The combination of HbA1c and FPG was not recommended as the first choice in diagnosing prediabetes.

### The ideal cutoff value of HbA1c for diagnosing DM and abnormal glucose level

A cutoff value of HbA1c of 6.3% was found in our data for diagnosing DM in our data, which is lower than that recommended by ADA (6.5%). This was similar to the reports from other studies [12,22]. The ideal cutoff value of HbA1c of 5.9% for diagnosing the overall abnormal glycometabolism in our study was slightly higher than that recommended by ADA (5.7%) and is different with that reported from Celik et al., (5.6%) [23]. The discrepancy may be due to the different study populations.

### Merits and limitations

The ACROSS-China study is a multi-central, nationwide prospective cohort study. To our knowledge, it was the first time that OGTT was nationwide used to detect abnormal glucose among in-hospital patients with acute stroke in China. However, OGTT was performed on the 14th day after stroke onset and patients might receive medications, which might affect the test results. Since OGTT was used as the golden method in the present study, the results might be affected. The present study was only a cross-sectional study. The research methods were not repeated to assess the reproducibility of the findings. A much bigger sample size might improve the comparison between the AUROCs to better differentiate the diagnostic ability among these tools.

## Conclusions

The present study found that the three tests (HbA1c, FPG, and OGTT) identified different DM or prediabetes patients in subjects with acute ischemic stroke. The overlap in diagnosing abnormal glycometabolism between HbA1c and FPG, HbA1c and OGTT, or all three was low, respectively. Combining HbA1c and FPG increased the detection rate of DM, compared with OGTT. Our results advocate the use of HbA1c as screening tool for the diagnosis of pre-diabetes.

## Additional file

Additional file 1:
**Clinical characteristic of the three glycometabolism statues according to OGTT results.** This table describes the clinical characteristics among the three abnormal glycometabolic profiles diagnosed by OGTT. The mean age, FPG, HbA1c, white cell counts, insulin resistance index, and low-density lipoprotein were all higher in the DM group (P < 0.05 in comparison among the three groups). The differences of other variables among the groups were not statistically significant. The variables were compared through one-way analysis of variance, test χ2 or Fisher’s exact test. A two-tailed P <0.05 was considered statistically significant.

## Abbreviations

AUROC: The area under the receiver operating characteristic curve
DM: Diabetes mellitus
FPG: Fasting plasma glucose
HbA1c: Glycated hemoglobin
IFG: Impaired fasting glucose
IGT: Impaired glucose tolerance
OGTT: Oral glucose tolerance test
WHO: World Health Organization
